# Creatine supplementation and exercise in aging: a narrative review of the muscle–brain axis and its impact on cognitive and physical health

**DOI:** 10.3389/fnut.2025.1687719

**Published:** 2026-01-12

**Authors:** Nana Li

**Affiliations:** School of Physical Education, Henan Polytechnic University, Jiaozuo, Henan, China

**Keywords:** creatine, exercise, cognitive function, aging, neuroprotection

## Abstract

**Background/Aim:**

Aging is associated with progressive declines in neuromuscular and cognitive functions, which negatively impact independence and quality of life. The muscle–brain axis has emerged as a key pathway linking skeletal muscle health to cognitive performance. This review aimed to evaluate the effects of creatine supplementation combined with exercise on physical and cognitive outcomes in older adults.

**Methods:**

A narrative review was conducted summarizing evidence from clinical and preclinical studies, including randomized controlled trials and meta-analyses, on creatine supplementation and exercise interventions targeting aging populations.

**Results:**

Creatine supplementation, particularly when combined with resistance training, significantly improves muscle strength, lean body mass, and functional capacity in older adults. Cognitive outcomes show modest improvements in memory, processing speed, and executive function, especially in individuals with lower baseline creatine levels. Mechanistically, creatine supports energy metabolism, mitochondrial stability, and antioxidant defenses, while exercise promotes neuroplasticity through myokines, collectively reinforcing the muscle–brain axis.

**Conclusion:**

The combination of creatine supplementation and structured exercise appears to be a safe and promising strategy to counteract age-related declines in both physical and cognitive functions. However, further large-scale studies are required to establish long-term benefits and optimize dosing protocols.

## Introduction

Aging is an inevitable process marked by progressive loss of physiological integrity which causes reduced function and increased vulnerability to disease (1). Aging significantly affects neuromuscular function, leading to declines in mobility, independence, and overall health (2, 3). This deterioration, commonly termed sarcopenia, involves loss of muscle mass, strength, and endurance, together with impairments in neuromuscular junctions and motor neuron activity (2, 3). These changes increase the risk of falls, fractures, and disability in older adults (2, 3). Notably, the decline in neuro-muscular function does not transpire in isolation but is intricately linked to the aging processes of the brain, which involve declines in cognitive capacities such as executive function, memory, and processing speed (4).

Cognitive decline commonly accompanies aging and affects memory, executive function, and processing speed. Maintaining cognitive abilities is essential for independence and overall quality of life in older adults (5, 6). Healthy aging involves maintaining functional capacity, not merely the absence of disease (7, 8).

Emerging empirical findings indicate the presence of a critical biological interface stated to as the muscle–brain axis, which encompasses the reciprocal communication pathways that connect skeletal muscle and cognitive function (9). During contraction, it releases myokines that cross the blood-brain barrier (BBB) and influence neuroplasticity, neurogenesis, and brain metabolism (10, 11). This communication supports cognitive health and may help protect against neurodegeneration.

Conversely, brain health affects motor neuron activity and neuromuscular coordination, both of which are vital for maintaining muscular performance (12, 13). Disruption of this two-way communication, the muscle-brain axis, may accelerate both muscle atrophy and cognitive decline with aging. The exploration of the muscle-brain axis presents novel opportunities for therapeutic interventions that concurrently address both neuro-muscular and cognitive deterioration. Therapeutic modalities that augment muscular functionality may yield advantageous outcomes for cerebral health and, conversely, such interventions may also benefit muscular integrity.

Notably, creatine supplementation has garnered significant scholarly attention owing to its contributions to energy metabolism and its potential neuroprotective attributes. Systematically examining these interventions within the framework of the muscle-brain axis may yield pioneering strategies to confront the intricate challenges associated with the aging process.

Given that muscle-derived signals influence neuroplasticity and, conversely, neural health shapes motor output, separating physical and cognitive outcomes would overlook the very nature of this biological system. For this reason, the review considers both aspects together, emphasizing shared pathways and synergistic effects of creatine and exercise across the axis. Indeed, while creatine and exercise have been reviewed separately, the combined effects of these interventions on the muscle–brain axis have not been synthesized in a unified framework. This review integrates mechanistic, preclinical, and human evidence to highlight how creatine may potentiate exercise-induced adaptations across both muscular and cognitive domains. By connecting these strands of research, the manuscript aims to provide a cohesive perspective that is currently missing from the literature. The goal is not to repeat established findings but to contextualize them within a shared biological model relevant to aging. Indeed, a specific goal of this review is to evaluate whether creatine supplementation enhances the adaptations induced by exercise. Accordingly, this review specifically evaluates whether creatine supplementation produces synergistic effects when combined with exercise, in comparison to creatine-only and exercise-only interventions in similar older adult populations.

## Creatine: biochemistry and physiological role

Creatine is synthesized endogenously from amino acids and stored primarily in skeletal muscle, where it supports rapid ATP regeneration. Supplementation increases intramuscular creatine and phosphocreatine stores, enhancing short-term energy availability (14, 15). Creatine serves primarily as a rapid energy buffer through the phosphocreatine system, supporting ATP regeneration during high-demand conditions (16). Nearly 95% of the total body creatine is sequestered in muscle tissue, where it plays a pivotal role in supporting contractile functions, particularly during brief episodes of high-intensity exertion.

Supplementation has been reported to augment intramuscular total creatine and PCr reservoirs, thereby enhancing energy accessibility, postponing fatigue onset, and ameliorating recovery processes (17). In the brain, creatine contributes to energy buffering, mitochondrial stability, and antioxidant defense, which may support cognition and resilience against age-related neurodegeneration (18, 19). These attributes have profound implications for the pathophysiology of neurodegenerative diseases, traumatic brain injury, and the cognitive decline associated with aging.

In the context of aging, creatine intake together with physical exercise may help preserve muscular performance and sustain physical activity levels. These benefits in turn foster neuroplasticity and cognitive well-being through the muscle–brain axis. Considering its dual functionality in muscle and cerebral energetics, creatine emerges as a promising therapeutic intervention to alleviate sarcopenia, sustain mobility, and improve the QoL in elderly populations while potentially diminishing the likelihood of neurodegenerative disorders (20). Only mechanisms relevant to aging and the muscle–brain axis are summarized here.

## The muscle-brain axis: concept and mechanisms

Mechanistic Basis (Myokines and Signaling): The muscle-brain axis delineates a bidirectional communication network wherein skeletal muscle exerts influence over brain health and cognitive performance via endocrine, paracrine, and neural mechanisms. Skeletal muscle operates as an endocrine organ, secreting exercise-induced biomarkers and peptides collectively referred to as myokines, which can interact with the brain to facilitate neuroprotection, synaptic plasticity, and cognitive resilience (9). Among the extensively investigated myokines is irisin, a cleavage product of fibronectin type III domain-containing protein 5 (FNDC5) that is upregulated by peroxisome proliferator-activated receptor gamma coactivator 1-alpha (PGC-1α) during muscle contraction; this myokine is capable of traversing the blood-brain barrier (BBB) and stimulating the expression of hippocampal brain-derived neurotrophic factor (BDNF), thereby augmenting synaptic functionality, neurogenesis, and memory (21). BDNF is a key mediator linking muscle activity to neuroplasticity. Exercise-induced BDNF release supports synaptic function and may contribute to cognitive benefits (22).

IGF-1 also participates in muscle–brain signaling and is modulated by physical activity (23). The health of skeletal muscle is intricately related to cognitive outcomes; elevated strength and muscle mass, exemplified by metrics such as handgrip or quadriceps strength, correlate with improved executive function, memory retention, and processing speed among older adults (24). Mechanistically, muscle contractions exert complex and context-dependent effects on inflammatory processes. While acute high-intensity contractions may transiently elevate pro-inflammatory cytokines, regular moderate exercise promotes an anti-inflammatory milieu through myokine release, improved mitochondrial efficiency, and enhanced neuromuscular junction integrity (25–28). These biochemical and endocrine interactions form the mechanistic foundation of the muscle–brain axis. The following preclinical and human studies illustrate how these pathways operate in practice.

Evidence from preclinical models: a study was conducted to determine the impact of aerobic exercise on the progression of Alzheimer's disease (AD) in APP/PS1 mice via the regulation of mitochondrial proteostasis. Mice were systematically categorized into distinct control and exercise subgroups characterized by varying states of mitochondrial activity and subjected to a 12-week training regimen on a treadmill. Behavioral assessments, including the Morris water maze and the step-down test, demonstrated that aerobic exercise markedly enhanced learning and memory capabilities, particularly in cohorts exhibiting activated mitochondrial functionality, while inhibition led to deleterious outcomes. Molecular examinations indicated that exercise elicited an elevation in the mitochondrial unfolded protein response and autophagy, concurrently diminishing mitochondrial protein import, thereby suggesting an augmentation of mitochondrial quality control mechanisms. Collectively, aerobic exercise facilitated improvements in cognitive function and postponed the manifestation of AD symptoms through the modulation of mitochondrial proteostasis (29). It is important to note that many of these findings are correlative rather than causal, underscoring the need for longitudinal and mechanistic studies. For example, associations between sarcopenia and cognitive impairment, or between exercise and neurogenesis, are strongly supported by observational and preclinical studies, but direct causal links in humans remain limited. Differentiating between causal pathways and correlative associations helps clarify where evidence is robust and where further mechanistic research is needed.

Evidence from human studies: findings in older adults further support the relevance of muscle–brain interactions across aging. A comprehensive meta-analysis investigated the correlation between sarcopenia and cognitive impairment in the geriatric population. Of the 274 studies identified, 10 fulfilled the inclusion criteria (comprising 9,703 participants), with 6 studies incorporated into the meta-analysis (encompassing 7,045 participants). The average prevalence rate of sarcopenia was determined to be 10.5%. Cognitive impairment was observed in 40% of individuals diagnosed with sarcopenia, in contrast to 25.3% of individuals without sarcopenia. The pooled analysis revealed that sarcopenia was significantly linked to increased odds of cognitive impairment, although the heterogeneity was notably high. These findings imply that sarcopenia could serve as a risk factor for cognitive decline, necessitating further longitudinal investigations to elucidate causality (30).

These findings underscore the significance of skeletal muscle as a critical regulator of cerebral function and position physical activity, alongside muscle-targeted interventions, as promising strategies for the prevention or mitigation of cognitive decline. While the muscle–brain axis framework is supported by a growing body of research, much of the evidence remains correlative. Established mechanisms include the role of creatine in phosphocreatine buffering and ATP regeneration, and exercise-induced increases in BDNF and IGF-1, which have been consistently demonstrated (9, 23, 31). In contrast, emerging pathways such as exosome signaling, mitochondrial proteostasis, and certain myokine-mediated effects remain more speculative and require validation in causal studies. Distinguishing between well-supported mechanisms and those that are still hypothetical is critical for interpreting current evidence and guiding future investigations. Noteworthy, most available findings linking muscle signaling, neurotrophic factors, and cognitive outcomes are correlational and often cross-sectional. To clarify directionality, future work should prioritize longitudinal cohorts with repeated mechanistic biomarkers, randomized exercise interventions that incorporate creatine supplementation arms, and experimental designs that manipulate muscle-derived signals such as BDNF or irisin. Studies combining neuroimaging, muscle phenotyping, and molecular profiling would also help establish causality and identify the specific pathways through which muscle activity influences cognitive aging.

### Creatine supplementation and muscle function in aging

Creatine supplementation has garnered substantial interest as a therapeutic intervention aimed at mitigating the age-associated deterioration of muscular function. The aging process is frequently linked to sarcopenia, which is denoted by a decrease in muscle mass, strength, and performance, thereby adversely influencing mobility, autonomy, and overall life quality. Creatine, a naturally occurring compound integral to cellular energy metabolism, serves a crucial function in the preservation of muscle performance via its role in the PCr energy system. The administration of creatine supplements has been demonstrated to augment muscle mass and strength in the elderly by elevating intramuscular PCr reserves, thus enhancing the capacity for rapid ATP regeneration during high-intensity exertions (32).

Empirical studies consistently indicate favorable outcomes of creatine intake in combination with resistance training on strength, muscle mass, and functional performance among geriatric populations. For instance, a meta-analysis conducted by Devries and Phillips (32) revealed that creatine intake yielded more significant increases in strength and lean mass relative to a placebo in older adults participating in resistance exercise. Additional randomized controlled trials have illustrated enhancements in muscle endurance, ambulation speed, and chair-rise capability, which are essential indicators of autonomy in older adults (33). Furthermore, the administration of creatine as a supplement is typically well-accepted, with a limited incidence of adverse effects documented, thereby substantiating its viability as a long-term strategy for older demographics (34).

Altogether, these observations emphasize the capacity of creatine supplementation to alleviate the decline of muscle mass related to aging, consequently improving physical performance and overall QoL. Considering the intricate interrelationship between muscular health and cognitive function via the muscle-brain axis, the advantageous effects of creatine on muscle may also play a function in indirectly safeguarding cognitive health in the elderly, further underscoring its significance in therapeutic applications (35).

Beyond muscle mass and strength, muscular power, the ability to produce force rapidly, is a crucial determinant of physical function in older adults. The age-related decline in power, termed powerpenia, often occurs earlier and has a greater impact on mobility, balance, and fall risk than loss of strength alone (36). Power depends on neuromuscular coordination and type II muscle fiber efficiency, which are highly sensitive to aging. Evidence indicates that creatine supplementation, particularly when combined with high-velocity or resistance training, enhances lower-limb power and the rate of force development in older adults (20, 37). Recognizing powerpenia therefore provides a more comprehensive understanding of how creatine and exercise interventions preserve independence and functional capacity in aging populations.

### Creatine supplementation and cognitive function

Although creatine is well known for its muscular effects, its influence on cerebral energy metabolism and neuroprotection is mediated through the same mechanisms that support muscle function. Thus, cognitive outcomes are discussed within the same framework rather than as a separate topic

Creatine intake has garnered increasing scholarly attention regarding its potential to augment cognitive performance, especially within the realms of attention, memory, and processing speed, while concurrently piquing interest in its neuroprotective capabilities during the aging process. A meta-analysis encompassing 16 trials (*n* = 492; ages 20.8–76.4) assessed the impacts of creatine monohydrate (CrM) intake on cognitive function among adults. The outcomes indicated notable enhancements in memory (SMD = 0.31), attention time (SMD = −0.31), and processing speed (SMD = −0.51), albeit no significant impacts were observed on overall cognitive or executive functioning. The advantages were particularly evident among participants with pre-existing health conditions, individuals aged 18–60 years, and female subjects, with no discernible differences observed between short- and long-term intervention durations. The quality of evidence was assessed as moderate for memory-related outcomes and low for other cognitive domains. These findings imply that creatine supplementation may facilitate improvements in specific cognitive functions, particularly memory, attention, and processing speed; however, further large-scale, high-quality trials are requisite to substantiate and elucidate its effects and underlying mechanisms (38).

Another meta-analysis encompassing 10 trials investigated the impact of creatine intake on cognitive memory in individuals without health impairments. Collectively, creatine was found to significantly enhance memory performance in comparison to a placebo (SMD = 0.29), demonstrating a marked advantage in older adults aged 66–76 years (SMD = 0.88), whereas no significant effect was observed among younger participants aged 11–31 years (SMD = 0.03). The observed effects were not contingent upon dosage (approximately 2.2–20 g/day), duration of intervention (ranging from 5 days to 24 weeks), sex, or geographic location. These results imply that creatine intake may positively influence memory capabilities, especially in older populations (39). Importantly, both reviews also emphasized that the overall certainty of evidence remains limited. Xu et al. (38) rated memory outcomes as moderate quality but attention and processing speed as low quality, while Prokopidis et al. (39) similarly concluded that the evidence base is of low-to-moderate certainty. These assessments reflect recurring limitations, including small sample sizes, short interventions, and inconsistent methodologies, which should temper interpretation of the findings.

In elderly individuals (68–85 years), a regimen of high-dose, short-term creatine loading (20 g/day for 7 days) was beneficial in enhancing recall and long-term memory (40), and supplementation in vegetarians (5 g/day for 6 weeks) enhanced working memory (41). Furthermore, creatine has been demonstrated to mitigate cognitive deficits resulting from sleep deprivation, particularly in the context of challenging executive tasks (40).

A comprehensive narrative review on the supplementation of CrM among older adults has determined that CrM is deemed safe and, particularly when synergistically administered with physical exercise, enhances lean body mass (LBM), muscle hypertrophy and strength, as well as bone area and density, functional capacity, glucose metabolism, cognitive function, and memory retention. These observed advantages imply prospective applications in the management of age-associated sarcopenia, osteoporosis, physical frailty, and various metabolic or neuromuscular disorders (42).

Research conducted on animal models provides robust evidence supporting the neuroprotective effects of creatine. In aged C57Bl/6J murine models, the administration of creatine was found to extend the median healthy lifespan by approximately 9%, enhance neurobehavioral performance, decrease markers of oxidative stress, and upregulate genes associated with neuroprotection and cognitive learning (43). Experimental investigations into neurodegenerative conditions, including Huntington's disease, Parkinson's disease (PD), and amyotrophic lateral sclerosis, elucidate creatine's properties as anti-apoptotic, anti-excitotoxic, and antioxidant (44, 45). In individuals afflicted with creatine deficiency syndromes, supplementation has been shown to partially restore cognitive functions (46). Preliminary clinical investigations concerning PD indicated transient symptomatic advantages; however, extensive longitudinal studies revealed no substantial influence on the trajectory of the disease (47).

Taken together, empirical evidence derived from human studies corroborates modest yet persistent enhancements in memory function, particularly among the elderly or during periods of cognitive strain, whereas research conducted on animal models robustly affirms neuroprotective effects through mechanisms related to cellular bioenergetics and the modulation of oxidative stress. However, not all clinical investigations have demonstrated cognitive benefits. For example, studies in older women, middle-aged men, and resistance-trained adults (48–50) reported no significant improvements in cognitive outcomes despite gains in muscular performance. These discrepancies likely reflect differences in participant demographics, baseline cognitive status, intervention duration, and the sensitivity of cognitive tests employed.

This inconsistency highlights the importance of larger, standardized RCTs with harmonized protocols to clarify the true scope of creatine's neurocognitive potential. Another limitation is the generally small sample sizes of many clinical trials, often ranging from fewer than 10 to fewer than 50 participants, which restricts statistical power and increases the risk of type II error. Although meta-analyses provide a broader perspective, the overall quality of evidence for creatine's effects on cognition is typically rated as moderate to low (38, 39). This indicates that while preliminary results are promising, the certainty of conclusions remains limited and further large-scale, high-quality RCTs are required.

## Synergistic effects: creatine + exercise vs. creatine alone or exercise alone

Across available trials, combined creatine plus exercise interventions produced greater improvements in muscle strength, lean mass, and functional capacity than creatine-only or exercise-only conditions in comparable older adult samples. Studies directly comparing all three conditions consistently showed:

- larger increases in strength (20, 37, 51),
- greater gains in lean mass (33, 51),
- and more pronounced improvements in lower-limb power (20).

These findings support a synergistic interaction between creatine and resistance training rather than an additive effect.

## Exercise as a therapeutic modality

Regular physical activity is one of the most effective non-drug strategies to counter age-related declines in muscle and cognitive health. Different types of exercise provide complementary benefits for older adults. Aerobic exercise modalities, including brisk walking, cycling, or swimming, are known to enhance cardiovascular fitness, augment cerebral blood flow, increase hippocampal volume, and facilitate neurogenesis through elevated concentrations of BDNF and vascular endothelial growth factor (52).

Resistance training, which targets major muscle groups at moderate to high intensity [60–85% of one-repetition maximum (1RM)], contributes to the protection of muscle mass, enhancement of strength and functional mobility, and has been correlated with improvements in executive function, memory, and processing speed (53, 54). Balance and flexibility training modalities, including Tai Chi, yoga, and specific stability exercises, are essential for mitigating fall risk, preserving proprioceptive abilities, and facilitating neuromuscular coordination, particularly among frail elderly populations (55).

Comprehensive training regimens that amalgamate aerobic, resistance, and balance exercises demonstrate markedly superior effects on both physical and cognitive performance when contrasted with singular modality approaches, especially when these interventions are maintained for durations of six months or more (56). The physiological effects of exercise on muscle tissue manifest through the activation of protein synthesis via the mTOR signaling pathway, enhancement of mitochondrial functionality, augmentation of glucose uptake through the translocation of GLUT4, and a reduction in intramuscular adipose tissue infiltration, all of which serve to combat sarcopenia and metabolic impairments (57).

In the cerebral context, physical activity promotes synaptic plasticity, increases the volume of gray matter, modulates neurotransmitter systems, and enhances cerebrovascular regulation, collectively bolstering cognitive function, emotional well-being, and resilience against neurodegenerative processes (58).

Resistance training, specifically, has been correlated with elevated levels of circulating IGF-1 and reduced homocysteine concentrations, mechanisms that may elucidate cognitive enhancements in the aging population (59). When integrated with creatine supplementation, physical exercise may yield synergistic effects through the muscle–brain axis. Creatine augments PCr reservoirs in both skeletal muscle and the cerebral context, facilitating ATP regeneration during tasks characterized by high energy demands, thereby enhancing muscular performance and potentially improving neural energy metabolism (60). Resistance training escalates the necessity for rapid ATP resynthesis, a biochemical process in which creatine is pivotal, potentially amplifying strength and hypertrophy outcomes (60). Within the CNS, the neurotrophic signaling induced by exercise, in conjunction with creatine's function in cellular energy buffering, may engender more significant advancements in cognitive function than either intervention could achieve independently (61).

Numerous investigations have documented additive or even synergistic influences on functional capacity, fatigue resilience, and cognitive performance when creatine supplementation is concomitantly administered alongside structured exercise regimens in the geriatric population (33, 37). This synergistic approach signifies a promising therapeutic strategy aimed at alleviating age-associated declines in both muscular and cognitive faculties, underscoring the imperative for additional trials that explicitly focus on the integrated muscle–brain advantages within the elderly demographic.

## Safety, dosage, and creatine practical considerations

### Exercise guidelines for aging populations

The integration of creatine supplementation with systematic and structured physical exercise enhances the advantages via the muscle–brain connection. Global recommendations for elderly individuals advocate for participation in 75–150 min of vigorous exercise or a minimum of 150–300 min weekly of moderate-intensity aerobic exercise, complemented by muscle-strengthening activities that engage all principal muscle groups on two or more occasions each week (62). Moreover, balance and flexibility training ought to be conducted at least thrice weekly to mitigate the risk of falls (55).

Resistance training at intensities ranging from 60 to 85% of 1RM, executed two to three times weekly, has been demonstrated to bolster muscle strength, maintain LBM, and enhance executive cognitive functions in the elderly population (54). Aerobic exercise modalities, including brisk walking, cycling, or swimming, promote the synthesis of BDNF, facilitate hippocampal neurogenesis, and enhance cognitive processing speed and memory retention (52). Multicomponent exercise interventions that integrate aerobic, resistance, and balance training for a minimum duration of six months demonstrate significant efficacy in preserving physical function, mitigating the onset of sarcopenia, and improving cognitive performance within aging demographics (56).

## Potential side effects and contraindications

The adverse effects related to creatine intake are predominantly mild and encompass transient water retention, increases in body mass, gastrointestinal discomfort, and muscle cramps, particularly when large single doses are ingested without sufficient hydration (17). While elevations in serum creatinine levels may be observed, these typically do not signify compromised renal function in otherwise healthy individuals (63). Longitudinal safety studies extending up to five years in healthy adults indicate no adverse impacts on renal or hepatic function when dosages recommended by health professionals are adhered to (64). Contraindications for creatine supplementation include pre-existing renal or hepatic disorders, necessitating avoidance or stringent monitoring in such populations. Adolescents and individuals below the age of 18 should refrain from supplementation without medical supervision due to the scarcity of safety data (65). Assurance of product quality through third-party certification is essential to mitigate risks associated with contamination. Another key issue for clinical translation is the lack of consensus on optimal dosing strategies. While typical maintenance doses of 3–5 g/day are widely recommended, some studies have tested higher intakes (10–20 g/day) with variable outcomes, and it is unclear whether certain subgroups require different dosing. Evidence also suggests substantial inter-individual variability, with “responders” and “non-responders” differing in baseline creatine levels, dietary intake, muscle fiber composition, and genetic factors. This variability complicates uniform recommendations and highlights the need for personalized approaches. Regarding long-term safety, available data up to five years in healthy adults indicate no adverse effects on renal or hepatic function at recommended doses. However, longitudinal studies in older populations and those with comorbidities remain limited, and more research is required to establish long-term safety profiles in diverse clinical settings. Another important aspect is the presence of responders and non-responders to creatine supplementation. Individual differences in baseline muscle creatine content, habitual dietary intake, muscle fiber composition, and genetic variation in the creatine transporter (SLC6A8) can all influence the degree of benefit observed. For instance, individuals with lower baseline creatine levels (such as vegetarians) often experience greater gains, while those with already high intramuscular creatine may show minimal response. Recognizing these patterns is essential for moving toward more individualized supplementation strategies in both research and clinical practice.

## Recommended dosage, administration, and safety considerations of creatine

CrM supplementation is typically acknowledged as safe for elderly populations when administered at appropriate dosages. A widely accepted protocol contains of an preliminary loading phase of approximately 0.3 g/kg/day (~20 g/day) for a duration of 5–7 days, succeeded by a maintenance dosage of 3–5 g/day (66). An alternative approach involves the exclusion of the loading phase, opting instead for a daily intake of 3–5 g for approximately four weeks, which results in comparable muscle creatine saturation without the rapid increase in body mass that is frequently observed with loading (35). For aging demographics, particularly when aiming to achieve both muscular and cognitive enhancements, maintenance doses of 3–5 g/day are extensively endorsed (17, 60). Certain clinical investigations examining brain creatine uptake and cognitive outcomes among older subjects, including individuals exhibiting mild cognitive injury, have safely administered elevated doses of 10–20 g/day over several weeks, observing significant increases in brain creatine concentrations (67), although such elevated dosages should be undertaken with medical oversight. Weight-based dosing of ~0.10–0.14 g/kg/day has also been proposed as a personalized approach to account for differences in body composition and absorption efficiency in the elderly (38).

## Cognitive function improvements with creatine and exercise

**Figure 1:**
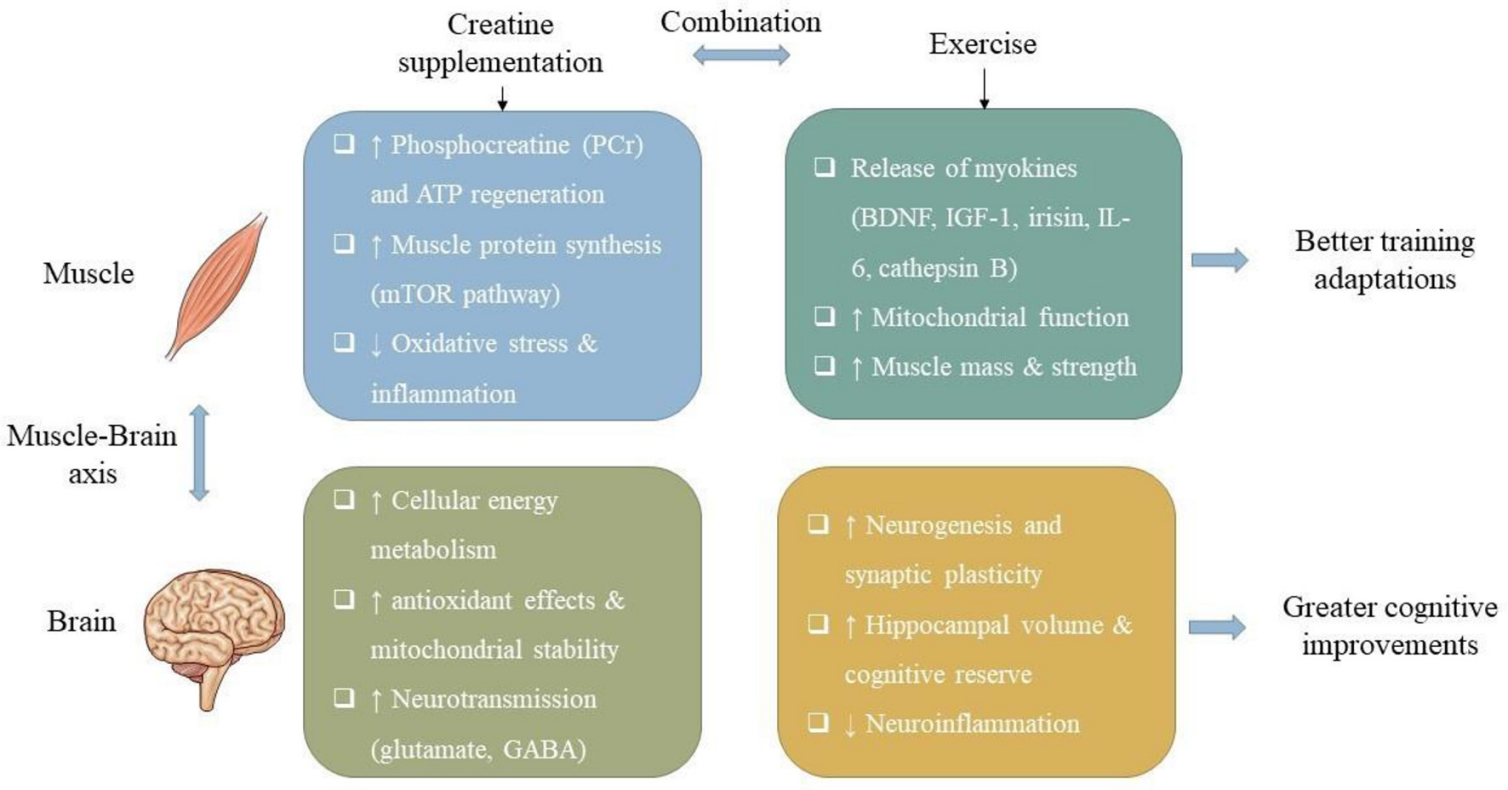
Beneficial effects of creatine supplementation and exercise on the muscle–brain axis. Creatine enhances phosphocreatine buffering, protein synthesis, and antioxidant defenses, while exercise promotes myokine release, mitochondrial function, and muscle adaptations. Together, these processes improve energy metabolism, neurotransmission, synaptic plasticity, and neurogenesis, resulting in better training adaptations and greater cognitive improvements. QoL, quality of life; BDNF, brain-derived neurotrophic factor.

The administration of creatine as a supplement, in conjunction with resistance training, has been shown to enhance physical functionality and muscular strength in elderly populations, thereby promoting increased autonomy in everyday activities. Enhanced muscular strength diminishes the likelihood of falls and aids in the preservation of mobility, indirectly fostering cognitive health by facilitating a more vigorous lifestyle. Furthermore, creatine intake has the possible to augment cognitive performance through the optimization of cerebral energy metabolism, particularly in contexts of stress or age-associated decline. Physical exercise fosters neuroplasticity and elevates the levels of neurotrophic factors like BDNF, which are instrumental in supporting cognitive domains such as attention, memory, and executive functioning. Additionally, strength training and physical activity have been demonstrated to alleviate depressive symptoms and enhance mood and self-efficacy in older subjects, thereby contributing to improved psychosocial well-being and overall life satisfaction (Figure 1).

An investigation was conducted to assess the effects of a 12-week regimen of advanced resistance exercise targeting the lower limbs on hippocampal neurometabolites, blood biomarkers, and hippocampal volume in elderly individuals exhibiting varying levels of risk for mild cognitive impairment (MCI). Seventy cognitively healthy subjects, aged between 60 and 85 years, were classified according to their cognitive risk as determined by the Montreal Cognitive Assessment. Notable findings revealed elevated kynurenine levels and diminished subiculum volumes in participants classified as having a higher risk for MCI. Furthermore, alterations in the volume of the hippocampal CA1 region attributable to exercise were found to be inversely correlated with changes in the ratios of neurometabolites. These findings provide valuable insights into the differential impacts of resistance training on biomarkers of brain health contingent upon the risk of MCI (68) (Table 1).

**Table 1:** Summary of creatine supplementation combined with resistance training on cognitive function for healthy aging.

**Participants (*n*; characteristics)**	**Creatine dose/supplement**	**Exercise protocol**	**Control group**	**Key cognitive findings**	**Ref**
70 older adults (60–85 y), stratified by MCI risk	None (exercise-only trial)	Lower-limb resistance training, 3 × /wk	Sedentary	High-risk group: ↑ kynurenine, ↓ subiculum volume; CA1 volume change inversely correlated with neurometabolite ratios	(68)
49 healthy older adults (mean age 73 y)	Multi-ingredient supplement incl. creatine (2.5 g/d), whey, n-3 PUFA, vitamin D, calcium	6 wk supplement → 12 wk resistance + HIIT	Supplement only	No cognitive effect with supplement alone; with exercise: ↑ MoCA, ↑ recall, ↓ reaction time	(70)
56 older women (4 groups)	Loading 20 g/d × 5 d → 5 g/d maintenance	Strength training ± creatine	Placebo, creatine-only, training-only	Training ↓ depression, ↑ strength; creatine no effect; no cognitive improvement	(48)
26 older adults (5M/21F)	Creatine 5 g/d	Resistance training	RT only	↑ Handgrip strength; ↑ MoCA score	(71)

RT, resistance training; 1RM, one-repetition maximum; MCI, mild cognitive impairment; MoCA, Montreal cognitive assessment.

Another investigation, the authors possess considerable engagement in the domain of creatine-related scholarship, commercial enterprises, and advisory capacities. DB directs a research and development initiative focused on creatine and has fulfilled the roles of product manager and scientific consultant, additionally receiving honoraria for participation in speaking engagements. SO co-holds a patent concerning liquid creatine supplements and has obtained research funding from a variety of organizations. DC and SF have executed industry-sponsored inquiries into creatine and have been recipients of product donations and support for academic presentations. JS boasts over 25 years of expertise in the ground of sports nutrition research and public speaking. RK has undertaken research on creatine, acted as an expert witness, and presides over a scientific advisory board pertaining to creatine. The majority of authors are members of this advisory board, which is funded by companies involved in creatine-related activities. The investigation was performed devoid of conflicts of interest, and the membership on the editorial board did not exert influence over the peer review or publication procedures (69).

A work evaluated the impact of a multi-ingredient supplement, administered with and without concomitant exercise, on cognitive functioning in a cohort of sedentary older males (mean age 73 years). A total of forty-nine male subjects were assigned to receive either the nutritional supplement (which comprised whey protein, omega-3 fatty acids, vitamin D, creatine, and calcium) or a placebo beverage over a duration of 20 weeks. The experimental design was bifurcated into two distinct phases: initially, a 7-week period involving either the supplement or placebo; subsequently, a 12-week regimen of resistance training and high-intensity interval training while maintaining the intake of the respective beverages. The results indicated an absence of cognitive enhancements following the administration of the supplement in isolation (Phase 1). Nevertheless, subsequent to the exercise intervention (Phase 2), participants exhibited significantly improved overall cognitive performance as measured by the Montreal Cognitive Assessment (MoCA), enhanced memory (specifically in word recall), and improved reaction times. Notable enhancements were predominantly observed within the supplement group, which correlated with elevated values of omega-3 fatty acids in the bloodstream. These findings imply that the synergistic combination of this nutritional supplement with physical exercise may serve to augment cognitive function in elderly males, potentially through the facilitation of improved omega-3 fatty acid bioavailability (70).

The 24-week investigation scrutinized the ramifications of creatine intake, with or without concomitant strength training, on emotional well-being and cognitive function among elderly female participants. The subjects were systematically allocated into four distinct cohorts: placebo, creatine, placebo coupled with strength training, and creatine accompanied by strength training. The findings indicated that the implementation of strength training, irrespective of creatine administration, markedly diminished depression scores and enhanced muscular strength when juxtaposed with non-trained counterparts. Nonetheless, creatine supplementation in isolation did not yield any significant alterations in emotional or cognitive metrics, and neither creatine nor strength training imparted any improvements in cognitive performance. Nutritional intake remained consistent throughout the study duration. In summary, creatine supplementation did not augment cognitive abilities or emotional health, whereas strength training was beneficial in enhancing mood and muscular strength, albeit without any concomitant enhancement in cognitive function, and did not confer additional advantages when combined with creatine (48).

Another 16-week longitudinal investigation evaluated the impact of resistance training in conjunction with creatine intake (5 g/day) on both cognitive function and muscular strength in a cohort of 26 elderly subjects. Participants were systematically assigned to either an experimental group (resistance training plus creatine) or a control group. Following the intervention, the cohort that engaged in resistance training accompanied by creatine demonstrated statistically significant enhancements in handgrip strength and cognitive performance (assessed via the MoCA) when compared with the control group. The findings of study indicate that this integrative strategy may facilitate improvements in both physical strength and cognitive capabilities among older people (71).

Overall, the administration of creatine in conjunction with resistance training exhibits promising advantages for the enhancement of physical capabilities, cognitive functioning, and emotional health in the elderly population. Although the isolated effects of creatine may be limited concerning cognitive or emotional states, its synergistic relationship with physical exercise significantly augments neuroplasticity and comprehensive cerebral health, thereby fostering autonomy and enhancing QoL in the context of aging. Additional investigations are warranted to refine intervention protocols and to elucidate the longitudinal effects.

## Targeting the muscle-brain axis and signaling pathways: combined creatine and exercise intervention

Creatine and physical exercise collaboratively augment the muscle–brain axis by facilitating energy metabolism and engaging critical signaling cascades. Creatine enhances ATP regeneration and mTOR-mediated protein synthesis within skeletal muscle, whereas physical exercise stimulates AMPK and PGC-1α, thereby advancing mitochondrial functionality. Collectively, these interventions elevate the release of myokines (e.g., BDNF, irisin), which fosters neurogenesis, synaptic plasticity, and cognitive performance, thereby establishing a nexus between enhanced cognitive advantages and muscular health in the context of aging.

**Figure 2:**
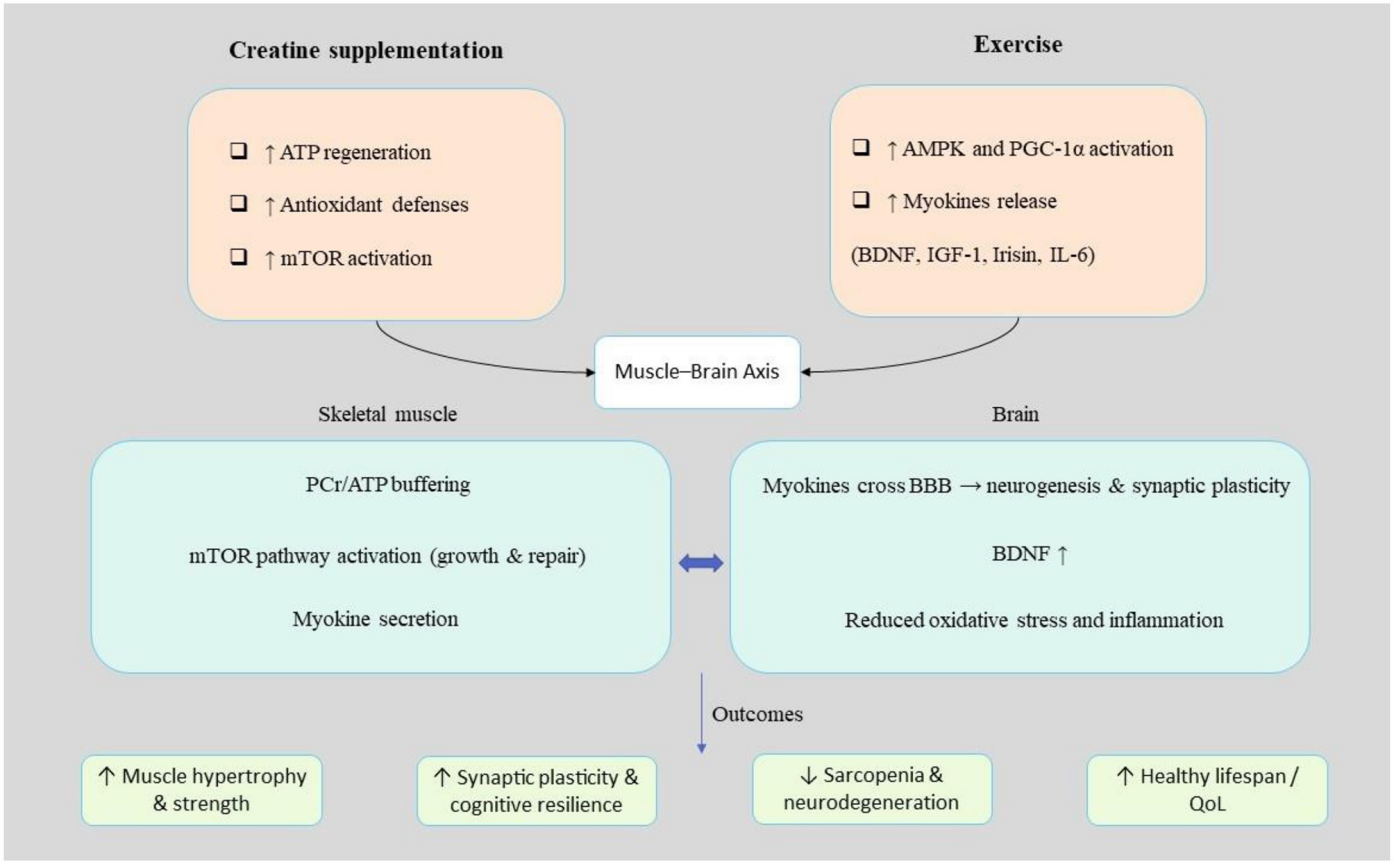
Targeting the muscle–brain axis through combined creatine and exercise intervention. Creatine supports ATP regeneration, mTOR pathway activation, and antioxidant defenses, while exercise induces AMPK/PGC-1α signaling and myokine release. Synergistically, these mechanisms enhance muscle strength, synaptic plasticity, and cognitive resilience, reducing sarcopenia and neurodegeneration and contributing to healthy lifespan and quality of life. MetS, metabolic syndrome; LBM, lean body mass.

Obesity and metabolic syndrome (MetS) adversely affect the anabolic response to protein supplementation and resistance training in older adults, thereby exacerbating the phenomenon of sarcopenic obesity (Figure 2). In a retrospective investigation involving 32 elderly male participants who engaged in a three-month regimen of home-based resistance training supplemented with protein-based multi-ingredient supplementation (MIS), the presence of obesity and MetS emerged as significant negative predictors of improvements in LBM, muscle-to-fat ratios, and strength, while variables such as physical activity levels, age, and renal function exerted minimal influence. A subgroup analysis that compared two distinct MIS formulations indicated that a whey/casein-based product (M5) facilitated more substantial enhancements in lean mass, strength, functional performance, and markers of bone turnover than a collagen-based formulation (PLA). Collectively, the anabolic response was markedly more pronounced in the M5 cohort. These results underscore the notion that obesity and MetS contribute to anabolic resistance in the aging population, emphasizing that high-quality protein intake combined with resistance training, proved to be more efficacious in maintaining musculoskeletal health among vulnerable older adults, notwithstanding adequate total protein consumption (72) (Table 2).

**Table 2:** Summary of creatine supplementation combined with resistance training on signaling pathways and muscle–brain axis for healthy aging.

**Participants (*n*; characteristics)**	**Creatine dose/supplement**	**Exercise protocol**	**Control group**	**Main outcomes (muscle–brain axis/signaling)**	**Ref**
12 older men with overweight/obesity + MetS risk	Multi-ingredient (protein, creatine, Ca, vit D3, fish oil)	Resistance training (home-based)	Supplement only	↑ Lean mass, ↑ strength, ↑ performance, ↑ bone markers	(72)
Aged male rats (*n* = 10/group)	Creatine 0.3 mg/kg	Resistance-type ladder climbing	Exercise only	↑ Lipid profile, ↑ antioxidants, ↑ neuromuscular markers, ↑ muscle histology	(73)
15 older adults (mean 68 y)	Creatine 0.1 g/kg/d	RT 3 × /wk	RT + placebo	↓ Oxidative stress, ↑ muscle strength, ↑ QoL	(74)
16 sedentary older men (mean 77 y)	Multi-ingredient (protein, creatine, vit D, omega-3)	Home-based RT	Supplement only	↑ Lean mass, ↑ leg & grip strength, ↓ Sit-to-Stand time, ↑ fiber CSA	(75)
Untrained aging men	Creatine 0.1 g/kg/d	High-velocity RT	HVRT only	↑ Lower-body strength, ↑ muscle thickness, ↑ balance	(76)
Elderly adults (RCT)	Low-dose creatine (dose NR)	RT 3 × /wk	Placebo + RT	↑ Lean mass vs. placebo; no difference in strength or BMD	(77)
Frail older adults (RCT)	Whey + creatine	RT (supervised)	Whey-only + RT	↑ Function (grip, TUG, sit-to-stand); no body comp change	(78)
60 older women (4 groups)	Creatine (dose per protocol)	RT	Placebo; RT-only	↑ Strength, ↑ appendicular lean mass	(51)
11 men (48–72 y)	Creatine 5 g/d + whey 35 g/d	RT	Whey only + RT	↑ Strength, ↑ lean mass	(49)
Older adults (6-month RT)	Creatine (dose NR)	RT 6 mo	RT + placebo	↑ Fat-free mass, ↑ strength	(79)
42 middle-aged men (4 groups)	Creatine 5 g in Gatorade	RT	Placebo + RT	↑ Arm/total lean mass, ↑ body water	(50)
42 middle-aged men (duplicate arm)	Creatine 5 g + whey 35 g (in Gatorade)	RT	Whey-only + RT	↑ Lean mass, ↑ body water	(50)
39 older adults (>65 y)	Creatine 5 g + CLA 6 g	RT	CLA-only + RT	↑ Strength, ↑ lean mass, ↓ fat mass; safe	(80)
44 adults (55–84 y)	Creatine + RT	RT	RT only	↑ Strength, ↑ lean mass	(81)
44 adults (55–84 y)	Creatine + ginseng/astragalus + RT	RT	Ginseng/astragalus + RT	↑ Strength, ↑ lean mass, ↑ lipids, ↑ vigor	(81)
8 older men (73 y)	Creatine cessation (no dose)	Reduced RT volume	Creatine continuation	Strength & lean mass maintained; ↓ endurance	(82)
28 older adults (>65 y, RCT)	Creatine 5 g/d	RT	Placebo + RT	↑ Lean mass, ↑ strength, ↑ intramuscular creatine, ↑ functional tasks	(83)

CrM, creatine monohydrate; RT, resistance training; LBM, lean body mass; CLA, conjugated linoleic acid.

Sarcopenia is a degenerative loss of strength and skeletal muscle mass that impairs Sarcopenia, characterized as the progressive decline in muscle strength and mass associated with aging, significantly detracts from overall QoL. In a cohort of aged rats, the investigation assessed the expression of the myogenin and NUDT3 gene as potential diagnostic biomarkers, while also evaluating the effects of a combined resistance exercise regimen with CrM in contrast to individual therapeutic interventions (exercise, CrM, or CoQ10). Over a 12-week period, the synergistic application of exercise and CrM led to substantial enhancements in serum lipid profiles, antioxidant indicators, electromyographic readings, as well as the expression levels of NUDT3 and myogenin, alongside improvements in CK and markers indicative of the sarcopenic index, when juxtaposed with singular treatment modalities. The findings supported the efficacy of NUDT3 and myogenin as reliable diagnostic instruments, while the concurrent administration of CrM with exercise was shown to facilitate muscle regeneration, mitigate inflammatory responses, and more effectively improve sarcopenia compared to isolated therapeutic approaches (73).

A ten-week investigation involving elderly participants demonstrated that resistance training (RT), with or without the adjunct of creatine supplementation (CS), led to a significant reduction in oxidative stress indicators (MDA, 8-OHdG) and an enhancement of antioxidant defenses (GPX, TAC). Both modalities yielded enhancements in muscle strength and overall QoL, with creatine intake contributing to a more pronounced increase in strength compared to RT in isolation. Resistance training is recognized as an efficacious non-pharmacological approach to augment antioxidant capacity, muscle functionality, and general well-being in the aging population, while creatine supplementation may further magnify these advantageous effects (74).

In older male populations, a 12-week regimen of home-based resistance band training, supplemented with a multi-component nutritional formulation (Muscle5: comprising casein, vitamin D, whey protein, creatine, and omega-3 fatty acids), demonstrated statistically significant enhancements in LBM, muscular strength, functional performance, and the size of fast-twitch muscle fibers when compared to a placebo control group. The improvements were especially pronounced among participants exhibiting sarcopenia. Muscle5 represents a safe and efficacious complement to low-intensity resistance training aimed at augmenting muscle strength, mass, and overall quality in geriatric individuals (75).

In untrained elderly males, a duration of 8 weeks engaged in high-velocity resistance training (HVRT) resulted in a noteworthy enhancement in muscle hypertrophy, muscular strength, peak torque, and overall physical performance. The incorporation of creatine supplementation further amplified the improvements observed in leg press performance and aggregate lower-body strength. Both HVRT and creatine supplementation emerged as safe and efficacious methodologies for enhancing muscle mass and functional capacity in the aging people (76).

In geriatric populations, a 12-week regimen of low-dose creatine intake in conjunction with resistance training led to a statistically significant enhancement of LBM in comparison to resistance training conducted in isolation. Nevertheless, the advancements in muscle strength, body composition, and the mineral density/content of bone did not exhibit statistically significant disparities between the two groups (77). In elderly individuals with reduced physical resilience, a duration of 14 weeks of resistance training, accompanied by either isolated whey protein or a combination of creatine and whey protein, yielded comparable enhancements in muscular performance (as assessed by timed-up-and-go, handgrip strength, and timed-stands evaluations). No statistically significant alterations were detected in body composition or hematological parameters, and both intervention strategies demonstrated safety and were well-accepted by participants (78).

In people of vulnerable older women, a regimen of 24 weeks involving creatine intake combined with resistance training (CR+RT) caused more pronounced enhancements in muscle strength (measured via 1-RM leg press and bench press) and appendicular LBM compared with the placebo, creatine supplementation alone, or resistance training in isolation. There were no statistically significant alterations noted in fat mass, bone mass, or serum markers indicative of bone metabolism. The intervention demonstrated efficacy in augmenting muscle mass and functional capacity, yet it did not yield improvements in bone health (51).

In middle-aged and senior males (ages 48–72 years), a 14-week regimen of conventional resistance training led to significant enhancements in both muscular strength and LBM across all experimental cohorts. The administration of creatine, protein, or a combination thereof did not yield any supplementary advantages in comparison to the placebo group. All cohorts exhibited comparable improvements, suggesting that resistance training in isolation was adequate to facilitate increases in muscle strength and mass within this demographic (49).

The phenomenon of human senescence lead to in sarcopenia, which is defined by diminished muscle mass, decreased strength, and compromised mitochondrial functionality. Engagement in resistance training has been indicated to augment muscle hypertrophy, enhance strength, improve mitochondrial capacity, and mitigate oxidative stress in the elderly population. Supplementation with CrM has been demonstrated to amplify the increases in fat-free mass and strength resultant from resistance training, while the incorporation of conjugated linoleic acid (CLA) may further facilitate reductions in body fat. Additionally, resistance training may promote mitochondrial quality by elevating the levels of wild-type mitochondrial DNA (mtDNA) and minimizing deletions, presumably through the activation of satellite cells that rejuvenate the mtDNA reservoir within muscle fibers. Existing evidence indicates that CrM may further stimulate satellite cell activation and increase the number of myonuclei. Further investigation is warranted to ascertain whether these mitochondrial and muscular adaptations are potentiated by CrM in older adults and whether the resultant effects are sustained over an extended duration (79).

Another investigation examined the impact of whey protein and creatine, both individually and in conjunction, on body composition among middle-aged males participating in resistance training. A total of forty-two males were randomly allocated into four distinct groups, placebo, whey protein, creatine, and the combination of creatine and whey protein, and underwent a training regimen three times weekly for a duration of 14 weeks. Body composition (assessed via DXA) and body water variables (intracellular water, total body water, extracellular water) were evaluated prior to and following the intervention. The resistance training program resulted in notable enhancements in regional arm adiposity (reduction), arm bone-free fat-free mass (augmentation), total body fat-free mass, as well as intracellular and extracellular water levels. Nonetheless, no significant changes were observed among the supplementation groups; neither creatine, whey protein, nor their combined administration conferred additional advantages beyond the effects of resistance training alone. Nutritional intake remained consistent throughout the duration of the study. In the context of middle-aged males, supplementation with creatine and/or whey protein does not amplify the alterations in body composition that are induced by resistance training (50).

A research investigation assessed the potential efficacy of CrM and CLA in augmenting strength and ameliorating body composition among subjects aged >65 years engaged in resistance training. A total of thirty-nine participants completed a six-month regimen of resistance training and were randomly assigned in a double-blind fashion to intake either CrM (5 g/day) in conjunction with CLA (6 g/day) or a placebo. Resistance training yielded statistically significant enhancements across all metrics of functional capacity and strength. The cohort receiving CrM+CLA exhibited superior advancements in isokinetic knee extension strength, muscular endurance, fat-free mass, and a reduction in fat mass in comparison to the placebo group. Serum creatinine values were elevated in the CrM+CLA group; however, creatinine clearance, CK, and hepatic function remained within normal ranges. These findings suggested that supervised resistance training is both safe and efficacious for the elderly population, and that supplementation with CrM+CLA can further augment strength, muscular endurance, and enhancements in body composition beyond the effects of training alone (80).

The impact of dietary creatine alongside a botanical extract comprising astragalus and ginseng was assessed in a cohort of 44 adults aged between 55 and 84 years who were engaged in a 12-week strength training regimen. Subjects were allocated to one of 3 groups: those consuming creatine exclusively, those receiving creatine in conjunction with the botanical extract (CrBE), or a placebo group, while undertaking exercises including knee extension, lat pull down, leg press, bench press, biceps curl, and knee flexion for three sets of 8 to 12 repetitions, three times a week. The primary outcomes measured included 1RM strength, body composition as evaluated by blood lipid profiles, full-body DEXA scans, and mood states. Notable enhancements in strength and lean mass were observed across all groups, with superior improvements noted in the creatine and CrBE groups relative to the placebo, although no significant differences were detected between the creatine and CrBE groups. It is noteworthy that only the CrBE group exhibited enhancements in blood lipid levels and self-reported vigor, in addition to a greater decrease in body fat and a rise in bench press strength compared to the Cr group (81).

Another investigation evaluated the ramifications of discontinuing creatine supplementation in conjunction with a 12-week regimen of reduced-volume training (33% reduction) within a specific cohort of older males (n = 8, mean age 73 years) in contrast to a control group of 5 males (mean age 69 years) who did not receive creatine. Metrics for strength (1-repetition maximum), muscular endurance (maximum repetitions across 3 sets at 70–80% of 1-RM), and LBM were quantified prior to and following the intervention. No significant alterations in strength or LBM were detected, while a notable decline in muscle endurance was observed (7–21%), with comparable rates of deterioration noted across both cohorts. These data suggested that the cessation of creatine intake does not affect the rate of decline in endurance, strength, or LBM during a 12-week period of reduced-volume resistance training (82).

The investigation assessed the potential of CrM supplementation to augment strength and fat-free mass improvements concomitant with resistance exercise training in elderly individuals. A cohort of twenty-eight healthy females and males aged 65 years and above engaged in a comprehensive whole-body resistance training regimen thrice weekly over a span of fourteen weeks and were allocated randomly, employing a double-blind methodology, to either receive CrM (5 g/d + 2 g dextrose) or a placebo (7 g dextrose). The primary metrics of interest encompassed fat-free mass, total body mass, 1RM strength across distinct body segments, isometric knee extension strength, handgrip strength, dorsiflexion strength, chair stand performance, 30-meter walking test, 14-stair climbing performance, muscle fiber type and cross-sectional area, alongside intramuscular total creatine levels. The fourteen-week resistance training intervention resulted in statistically significant enhancements across all strength assessments, functional task execution, and muscle fiber cross-sectional area in both experimental groups. Notably, CrM supplementation resulted in more pronounced increases in fat-free mass and overall body mass relative to the placebo condition (83). Overall, creatine and physical exercise collaboratively optimize the muscle-brain axis, fostering improvements in muscle mass, strength, and cognitive performance during the aging process. Their synergistic impact on energy metabolism, protein synthesis, and myokine signaling underpins neuroplasticity and enhances overall QoL, underscoring a promising therapeutic avenue for mitigating age-associated muscular and cognitive deterioration.

## Limitations and conflicting evidence

Although the literature on creatine and aging is extensive, findings remain mixed. While some trials demonstrate improvements in cognition and muscle outcomes, others report null effects, particularly in older women and middle-aged men. Methodological heterogeneity, small sample sizes, and moderate-to-low evidence quality further limit certainty. These issues underscore the need to interpret current findings with caution and highlight the importance of more rigorous, standardized trials.

## Future directions and research gaps

Before considering future directions, it is important to recognize key methodological limitations in the current evidence. The reviewed studies differ substantially in dosing protocols (ranging from short-term loading strategies of 20 g/day to longer maintenance regimens of 2–5 g/day), intervention duration (5 days to 24 weeks), participant characteristics (age, sex, health status, and training background), and outcome measures (38, 39). Such variability makes it difficult to compare results directly and reduces the strength of pooled conclusions. In addition, many trials use small sample sizes and inconsistent control conditions, further limiting statistical power. Together, these methodological issues highlight the need for future research to adopt standardized protocols for dosing, duration, and validated outcome measures, alongside larger and more diverse cohorts to improve the reliability and generalizability of findings. Several gaps in the literature warrant emphasis. Recent high-quality RCTs have expanded understanding of creatine's effects, yet their findings remain underrepresented in current syntheses. More systematic integration of these trials is needed to clarify both muscular and cognitive outcomes. Additionally, dose–response relationships are poorly defined; while standard maintenance doses are effective for many, it is unclear whether higher or tailored dosing yields superior benefits in older adults or those with low baseline creatine.

Another underexplored area involves bioavailability and genetic variability. Polymorphisms in the creatine transporter gene (SLC6A8) and differences in dietary intake or gut absorption may influence individual responses, but these factors are rarely assessed in clinical studies. Addressing these gaps will be critical for refining evidence-based recommendations and moving toward personalized strategies. Despite considerable advancements in the comprehension of the muscle-brain axis and the therapeutic implications of creatine supplementation in conjunction with exercise, several critical domains necessitate further scholarly exploration. Emerging empirical evidence is commencing to elucidate innovative mechanisms that govern muscle-to-brain communication, including the roles of specific myokines, exosomes, and mitochondrial signaling pathways, which may unveil new targets for therapeutic intervention. Moreover, progress in the identification of biomarkers, encompassing circulating proteins, metabolites, and neuroimaging indicators, holds significant potential for enhanced monitoring of therapeutic responses and the trajectory of disease progression, thereby facilitating more individualized treatment paradigms.

Additionally, the discovery of novel therapeutic targets within these pathways could catalyze the formulation of more efficacious interventions that complement creatine and exercise or potentially serve as substitutes under particular circumstances. For instance, the modulation of inflammation, oxidative stress, or mitochondrial dysfunction via pharmacological or nutritional strategies may augment the advantages observed with existing methodologies. To augment the robustness of the evidence base, forthcoming clinical trials ought to be meticulously designed with larger participant cohorts, extended follow-up durations, and a more heterogeneous demographic, encompassing individuals with varying levels of cognitive impairment and physical frailty. The standardization of protocols pertaining to creatine dosage, types of exercise, and outcome metrics will significantly enhance the comparability across various studies.

Furthermore, the incorporation of multimodal assessments, integrating cognitive evaluations, muscle function assessments, and biomarker analyses, will facilitate a more holistic understanding of the effects these interventions exert on the muscle–brain axis and the overall QoL among aging individuals. Addressing these identified deficiencies will be pivotal for refining therapeutic strategies and for the effective translation of research outcomes into clinical practices aimed at enhancing cognitive health and physical functionality within the aging populace. To strengthen the evidence base, future research should adopt more rigorous design standards. Large, multi-center RCTs with adequate statistical power are needed to confirm both muscular and cognitive effects of creatine in aging populations. Standardized dosing protocols and intervention durations would improve comparability across trials. Trials should also incorporate stratification by sex, age, diet, and baseline creatine levels to better capture inter-individual variability. Additionally, consistent use of validated cognitive and functional outcome measures, along with long-term safety monitoring, will be essential. Such design improvements will help clarify dose-response relationships, establish causality, and guide the development of personalized supplementation strategies.

## Conclusions

Creatine supplementation and exercise each provide meaningful benefits for muscle strength, physical function, and cognitive health in older adults, but the emerging evidence indicates that their combination may produce advantages beyond either intervention alone. Studies that directly compare creatine-only, exercise-only, and combined protocols generally report greater gains in muscle performance, lean mass, and lower-limb power when the two interventions are applied together. These findings support a synergistic rather than additive interaction, aligning with the bidirectional nature of the muscle–brain axis and reinforcing the review's integrative perspective.

Although the biological mechanisms linking muscle and brain adaptations are increasingly understood, particularly the roles of energy metabolism, myokine signaling, and neurotrophic pathways, most available studies remain correlational, short in duration, or limited by small sample sizes. Future research should incorporate head-to-head trial designs that directly evaluate combined vs. isolated interventions, examine dose–response relationships for creatine in aging populations, and include mechanistic outcomes that clarify causality within the muscle–brain axis. Larger, longer-term studies with standardized cognitive and functional measures are also needed.

Overall, synthesizing creatine and exercise within the framework of the muscle–brain axis provides a clearer understanding of how these interventions intersect and where their combined application may offer the greatest clinical value. This perspective highlights a promising avenue for strategies aimed at preserving both physical and cognitive function in aging.
